# Identification of FADS2 as a Contributor of Ferroptosis Escape in Bladder Cancer

**DOI:** 10.1111/jcmm.70710

**Published:** 2025-07-19

**Authors:** Peixin Li, Shengwen Yao, Wenqiang Qi, Hanwen Liu, Xiaoyi Zhang, Bin Zhou, Shijie Zhang, Yaozhong Zhang, Hao Liang, Huangwei Huang, Yihao Zhao, Benkang Shi, Jun Chen, Jingchao Liu

**Affiliations:** ^1^ Department of Urology Qilu Hospital of Shandong University Jinan Shandong China; ^2^ Department of Urology The Second Hospital of Shandong University Jinan Shandong China; ^3^ Department of Medicine Jacobi Medical Center/Albert Einstein College of Medicine Bronx New York USA; ^4^ Department of Urology Qilu Hospital of Shandong University Qingdao Shandong China

**Keywords:** biomarker, bladder cancer, FADS2, ferroptosis, prognosis

## Abstract

Ferroptosis is an iron‐dependent form of regulated cell death. Previous research indicates that inducing ferroptosis holds significant promise in cancer therapy, particularly for patients who have failed traditional treatments. However, the presence of a ferroptosis escape mechanism in bladder cancer remains unclear, and the therapeutic potential of ferroptosis induction in this context requires further exploration. In this study, bioinformatics analyses and immunohistochemical staining revealed that FADS2 is aberrantly overexpressed in bladder cancer, with its high expression correlating with poor prognosis. Both in vivo and in vitro experiments, including CCK‐8 assays, lipid peroxidation assays, iron measurements and ferroptosis‐related gene analyses, demonstrated that silencing FADS2 can trigger ferroptosis in bladder cancer cells. Mechanistically, inhibition of the mTOR pathway and SREBP activity was found to reduce FADS2 expression and promote ferroptosis in bladder cancer. In conclusion, this study identifies a critical gene involved in ferroptosis escape in bladder cancer and suggests that FADS2 could serve as a novel prognostic marker and therapeutic target.

**Abbreviations**
CCK‐8: cell counting kit‐8
DMSO: dimethyl sulphoxide
FADS2: fatty acid desaturase gene 2
FASN: fatty acid synthase
GPX4: glutathione peroxidase 4
GSH: glutathione
IHC: immunohistochemistry
MDA: malondialdehyde
mTOR: mammalian target of rapamycin
ROS: reactive oxygen species
SREBP: sterol‐regulatory element binding protein
SCD: stearoyl‐coenzyme A desaturase
SLC7A11: solute carrier family 7 member 11
TCGA: The Cancer Genome Atlas

## 1 Introduction

Bladder cancer ranks among the most prevalent malignant tumours of the urinary system. According to 2022 epidemiological data, there were 613,791 new cases and 220,349 deaths, representing a significant threat to public health [1]. Bladder cancer is classified into nonmuscle‐invasive bladder cancer (NMIBC) and muscle‐invasive bladder cancer (MIBC) based on tumour infiltration depth. Despite effective treatments such as transurethral resection for patients diagnosed with NMIBC, 31%–78% of these individuals experience tumour recurrence within 5 years, and 10%–15% progress to MIBC. The 5‐year survival rate for patients with bladder cancer is only 36%–48% [2]. Platinum‐based combination chemotherapy remains the first‐line treatment for MIBC and metastatic bladder cancer. However, chemotherapy resistance and severe side effects limit its effectiveness, with only 40% of patients with MIBC benefiting from it. For metastatic bladder cancer, the 5‐year survival rate under chemotherapy ranges from 2.6% to 10% [3]. In summary, bladder cancer is marked by high rates of recurrence, progression and metastasis, necessitating a more profound understanding of its molecular mechanisms to identify novel therapeutic targets and improve patient survival.

Ferroptosis is an iron‐dependent form of programmed cell death first introduced by Dixon in 2012 [4, 5, 6, 7, 8]. Unlike traditional cell death mechanisms such as necrosis, apoptosis and autophagy, ferroptosis is characterised by disruptions in iron metabolism, lipid metabolism and antioxidant systems [8, 9, 10, 11, 12]. Subcellularly, it is characterised by rupture of the outer mitochondrial membrane and a reduction or loss of mitochondrial cristae [13, 14]. Tumour cells, which require increased iron for proliferation, are more susceptible to iron‐driven necrosis. Accumulating evidence indicates that ferroptosis plays a pivotal role in the onset and progression of several cancers, including hepatocellular carcinoma, colorectal cancer, breast cancer and nonsmall cell lung cancer [15, 16, 17, 18]. Consequently, ferroptosis induction represents a promising therapeutic strategy for bladder cancer, particularly in patients resistant to chemotherapy or ineffective immunotherapy. However, tumour cells can evade ferroptosis by reducing lipid peroxidation or enhancing antioxidant defence mechanisms [19, 20]. Thus, identifying key genes involved in ferroptosis resistance in bladder cancer is critical.

FADS2, a member of the fatty acid desaturase family, is a key enzyme in the synthesis of polyunsaturated fatty acids (PUFAs), playing a vital role in tumour progression and chemotherapy resistance [21, 22]. The fatty acid desaturase genes (FADS1, FADS2, FADS3) located on chromosome 11q12‐13.1 are responsible for the biosynthesis of highly unsaturated fatty acid precursors [23]. FADS2 is aberrantly overexpressed in several cancers, including ovarian, prostate and lung cancers [24, 25, 26]. In previous bioinformatics analyses [27, 28], FADS2 was identified as a ferroptosis‐related gene in bladder cancer. Although prognostic prediction models based on ferroptosis‐related genes in bladder cancer have been developed [29], the specific role of FADS2 in this context remains unclear. This study, therefore, aims to investigate whether FADS2 contributes to ferroptosis resistance in bladder cancer and whether it could serve as a novel therapeutic target and prognostic biomarker.

## 2 Materials and Methods

### 2.1 Data Collection and Processing

The transcriptome and clinical data of 430 bladder cancer samples were downloaded from The Cancer Genome Atlas (TCGA) (https://portal.gdc.cancer.gov/repository). All data were standardised using the Bioconductor ‘limma’ package (https://www.bioconductor.org/) in R version 4.2.0 (https://www.r‐project.org/). A total of 409 tumour samples with corresponding clinical information were used for univariate and multivariate Cox regression analyses and clinical relevance evaluations. All procedures in this study adhered to the guidelines and policies of the TCGA database.

### 2.2 Cell Culture

Bladder cancer cell lines SV‐HUC‐1, 5637, T24, UM‐UC‐3, BIU‐87 and HEK‐293 T were sourced from the Cell Bank of the Chinese Academy of Sciences (Shanghai, China). All cells were cultured in a humidified incubator at 37°C with 5% CO_2_, in appropriate media supplemented with 10% FBS (Gibco, USA), 100 μg/mL penicillin (Gibco, USA) and 100 μg/mL streptomycin (Gibco, USA).

### 2.3 Patient Specimens

Tissue samples of patients with bladder cancer, along with adjacent noncancerous tissues, were obtained from Qilu Hospital of Shandong University (Jinan, China), with ethical approval from the hospital's Ethics Committee. All clinical samples were collected with informed patient consent, and their use complied with the Helsinki Declaration's ethical guidelines for human subjects.

### 2.4 Reagents and Antibodies

Chemical inhibitors used in this study included Erastin (HY‐15763), Ferrostatin‐1 (HY‐100579), Necrostatin‐1 (HY‐15760), 3‐Methyladenine (HY‐19312), Z‐VAD‐FMK (HY‐16658B), Rapamycin (HY‐10219) and Fatostatin (HY‐14452), all purchased from MedChemExpress (MCE, USA). Antibodies used included Anti‐FADS2 (68026‐1‐Ig), anti‐GPX4 (67763‐1‐Ig), anti‐SLC7A11 (26864‐1‐AP), anti‐Ki67 (27309‐1‐AP, 1:6000) and Beta‐Actin (81115‐1‐RR), sourced from Proteintech (Wuhan, China).

### 2.5 Lentivirus Infection and Cell Transfection

Lentiviral infection was conducted to create a stable knockdown of FADS2 in bladder cancer cell lines. Briefly, the plasmid encoding the shRNA was cloned into a lentiviral vector, which was then transfected into HEK‐293 T cells. The resulting virus was used to infect bladder cancer cells, and puromycin selection was applied to isolate stable cell lines. For cell transfection, 3 × 10^5^ cells/well were seeded in 6‐well plates, and Lipofectamine 3000 (Invitrogen) was used to transfect the shRNA plasmids according to the manufacturer's protocol.

### 2.6 Small Interfering RNA Interference Assay

Small interfering RNA (siRNA) was purchased from GenePharma (Shanghai, China) and transfected into cell lines using Lipofectamine 3000 (Invitrogen, Carlsbad, CA, USA), following the manufacturer's instructions.

### 2.7 Transmission Electron Microscope (TEM) Sample Preparation and Observation

Cells were collected and fixed in 2.5% glutaraldehyde solution for 4 h at 4°C, followed by four washes with 0.1 mol/L phosphate buffer for 15 min each. The samples were then fixed with 1% osmium tetroxide solution for 2 h at 4°C and washed twice with 0.1 mol/L phosphate buffer for 5 min each. Dehydration was performed using a gradient of acetone (50%, 70%, 90% and 100%) for 15 min per concentration. For permeation, cells were treated for 2 h with a 1:1 mixture of 100% acetone and resin, then 2 h with a 1:2 mixture, and finally overnight with pure resin. Embedding was carried out using Epon 812 resin, followed by polymerisation in an oven at 37°C for 12 h, 45°C for 12 h and 60°C for 48 h. Ultra‐thin sections (60–70 nm) were cut using an Ultracut R slicer (Leica), stained with uranyl acetate and lead nitrate, and examined under a Tecnai G2 Spirit Bio TWIN transmission electron microscope (FEI).

### 2.8 Quantitative Real‐Time PCR (qRT‐PCR) Analysis

Total RNA was isolated from cells using the Quick RNA Extraction Kit (AG21023, Accurate Biology, China) following the manufacturer's protocol. Reverse transcription polymerase chain reaction (RT‐PCR) was performed by reverse transcribing 1 μg of RNA into complementary DNA (cDNA) using an RT reagent kit (AG11706, Accurate Biology, China). Quantitative PCR was conducted using SYBR qPCR Master Mix (AG11701, Accurate Biology, China) in a real‐time PCR system (Lightcycler96, Roche, China). SYBR Green dye was used to monitor amplification of the target DNA during each PCR cycle, and the fluorescence signal was recorded in real time. The primers used in this study were as follows: FADS2 forward: 5′‐TTGCTACCTCTCAGGCCCAA‐3′, FADS2 reverse: 5′‐TGCTGGAAGTGGCGATGATT‐3′; GPX4 forward: 5′‐TGGACGAGGGGAGGAGC‐3′, GPX4 reverse: 5′‐GGGACGCGCACATGGT‐3′; SLC7A11 forward: 5′‐TGACTGGAGTCCCTGCGTAT‐3′, SLC7A11 reverse: 5′‐TGTTCTGGTTATTTTCTCCGACA‐3′; GAPDH forward: 5′‐GCACCGTCAAGGCTGAGAAC‐3′, GAPDH reverse: 5′‐TGGTGAAGACGCCAGTGGA‐3′; SREBP1 forward: 5′‐CAACACAGCAACCAGAAACTCAA‐3′, SREBP1 reverse: 5′‐GTCCTCCACCTCAGTCTTCAC‐3′.

### 2.9 Western Blot

Proteins were extracted from cells using RIPA lysis buffer (Beyotime, China) containing protease inhibitors (Beyotime, China) and phosphatase inhibitors (Beyotime, China). Protein concentration was determined using a BCA protein assay kit (Beyotime, China), and 30 μg of protein was separated by 10% SDS‐PAGE. The proteins were transferred to a nitrocellulose membrane, followed by incubation with 5% low‐fat milk for 1 h. The membrane was incubated with the appropriate primary antibodies at 4°C overnight. After washing with TBST three times, the membrane was incubated with horseradish peroxidase‐conjugated secondary antibody for 1 h at room temperature. Protein signals were detected using a chemiluminescent imaging system (Tanon 5200, Tanon, Shanghai, China).

### 2.10 Immunohistochemistry (IHC) Analysis

The paraffin‐embedded tissue sections were dewaxed, subjected to antigen retrieval, and incubated overnight with specific primary antibodies at 4°C. Subsequently, the tissue sections were incubated with biotinylated goat anti‐rabbit IgG for 20 min at room temperature, followed by incubation with streptavidin horseradish peroxidase for 30 min. Finally, tissues were stained with diaminobenzidine‐H_2_O_2_ and haematoxylin.

### 2.11 Mitochondrial Membrane Potential Staining

The cyanine dye JC‐1 was used as a fluorescent probe to assess mitochondrial membrane potential. Cells were incubated with JC‐1 at 37°C for 30 min and then washed for imaging using a fluorescence microscope (ZEISS, Germany) to visualise mitochondrial alterations.

### 2.12 Glutathione Assay

The GSH concentration in cells was measured using a GSH assay kit (HY‐K0311, MCE) according to the manufacturer's instructions, with absorbance recorded at 412 nm using a microplate reader.

### 2.13 Malondialdehyde Assay

Lipid peroxidation was assessed using a malondialdehyde (MDA) colorimetric assay kit (S0131S, Beyotime) following the manufacturer's protocol. Absorbance at 532 nm was measured, and MDA concentration was calculated based on the provided formula.

### 2.14 Intracellular ROS Measurements

Intracellular reactive oxygen species (ROS) levels were quantified using the following protocol: 1 × 10^6^ cells, either treated or transfected, were seeded in 6‐well plates and cultured for 24 h. The medium was then replaced with 1 mL of fresh serum‐free medium containing a suitable concentration of DCFH‐DA (D6470, Solarbio, China), and cells were incubated at 37°C for 30 min. After incubation, cells were washed three times with serum‐free medium to remove residual DCFH‐DA. ROS levels were observed by green fluorescence using a fluorescence microscope (ZEISS, Germany).

### 2.15 Lipid Peroxidation Assay

To assess lipid peroxidation, 1 × 10^6^ cells were seeded in a 6‐well plate. The following day, the medium was replaced with 1 mL of fresh FBS‐free medium containing 2 μM C11‐BODIPY 581/591 (D3861, Invitrogen, USA) and incubated at 37°C for 30 min. After washing the cells three times with PBS to remove residual probe, FITC fluorescence intensity was measured using a flow cytometer (CytoFLEX S, BECKMAN COULTER, USA), and mean fluorescence intensity was analysed using FlowJo.

### 2.16 Iron Assay

For ferrous ion measurement, mouse tumour tissue was homogenised and centrifuged at 12,000 rpm for 15 min. The supernatant was collected and diluted to one‐quarter of its original concentration. Ferrous ion concentration was determined using the ferrous ion colorimetric kit (E‐BC‐K773‐M, Elabscience, China), with absorbance measured at 597 nm and concentrations calculated using a standard curve.

### 2.17 Cell Counting Kit‐8 (CCK‐8) Assay

Cell proliferation was measured using the CCK‐8 assay. A total of 3000 cells per well were seeded in a 96‐well plate, with five replicates for each condition. After 24 h of incubation at 37°C, CCK‐8 reagent (CX001S, Epizyme, China) was added to each well (10 μL per 100 μL medium), and incubation continued for an additional 2 h. Absorbance was measured at 450 nm using a microplate reader (Tecan, Switzerland).

### 2.18 Colony Formation Assay

The colony formation assay was performed to evaluate the proliferative capability of individual cells. Briefly, 800 cells were seeded into each well of a 6‐well plate. Cells were cultured in a suitable incubator for 7–14 days, with medium replacement every 3 days. Following the incubation period, colony masses were fixed with 4% paraformaldehyde and stained with 0.5% crystal violet for 20 min.

### 2.19 Transwell Migration Assay

To assess cell migration, the transwell migration assay was conducted using a transwell chamber. Specifically, 1 × 10^4^ cells per well were resuspended in fresh medium without foetal bovine serum (FBS) and seeded in the upper chamber of the transwell. The lower chamber was filled with 700 μL of fresh medium containing 10% FBS. The chamber was incubated under suitable conditions for 24 h. After incubation, cells that migrated through the membrane into the lower chamber were fixed with 4% paraformaldehyde and stained with 0.5% crystal violet for 20 min. The migrated cells were then captured using an electron microscope (Olympus, Germany).

### 2.20 Wound Healing Assay

The wound healing assay was used to evaluate cell migration following transfection. Cells were seeded in 6‐well plates and cultured until they reached 90% confluence. A wound was made across the monolayer using a 200 μL pipette tip. The migration of cells into the wound area was recorded at 0, 12 and 24 h using an electron microscope (Olympus, Germany). The wound closure was quantified using image analysis software, such as ImageJ.

### 2.21 Mouse Xenografts

Animal experiments were approved by the Ethics Committee of Qilu Hospital of Shandong University. Female BALB/c nude mice (8 weeks old) were purchased from Beijing Vitalstar Biotechnology Co. Ltd. The experiment followed the Declaration of Helsinki and complied with China's ‘Guidance Suggestions on the Care and Use of Animals.’ Biu‐87 cells, with or without transfection, were subcutaneously implanted into the left armpit of the nude mice (*n* = 5 per group). The animals were sacrificed after 15 days, and the subcutaneous tumours were removed for subsequent analyses. Mice body weight, tumour diameter and volume were monitored every 2 days. Tumour volume was calculated using the formula: tumour volume (mm^3^) = tumour length × width^2^/2.

### 2.22 Statistical Analysis

Statistical analyses were performed using SPSS Statistics 26, GraphPad Prism 8, FlowJo v10.8.1 and ImageJ 1.45. A t‐test was used for comparisons between two or three groups, while the χ^2^ test was employed to evaluate clinical characteristics of patients. Survival curves were generated using the Kaplan–Meier method. Spearman's correlation was used to assess gene relationships. All data are presented as the mean ± SD from three independent experiments. A *p* value of less than 0.05 was considered statistically significant.

## 3 Results

### 3.1 FADS2 Expression Is Upregulated in BCa

**FIGURE 1:**
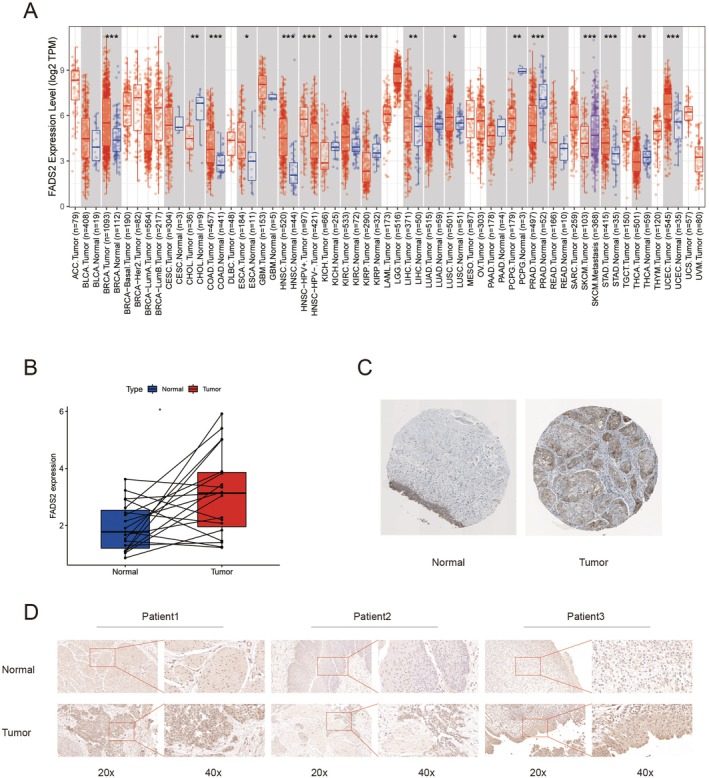
Expression of FADS2 in bladder cancer. (A) Pan‐cancer analysis from the TCGA database. (B) Paired differential analysis of FADS2 expression in bladder cancer tissues. (C) Immunohistochemical staining of FADS2 using data from the HPA database. (D) Immunohistochemical staining of FADS2 in bladder cancer tissues from patients.

Pan‐cancer analysis of the TCGA database demonstrated that FADS2 is overexpressed in various cancers, including bladder, breast, colon, oesophageal and kidney cancers (Figure 1A). In bladder cancer, paired differential analysis revealed significantly higher FADS2 expression in tumour tissues compared to adjacent noncancerous tissues (Figure 1B). Furthermore, immunohistochemical staining analysis from the HPA database also showed a marked increase in FADS2 expression in bladder cancer tissues (Figure 1C). To corroborate these findings, immunohistochemical staining of FADS2 was performed on pathological slices obtained from patients with bladder cancer at our centre, which confirmed the elevated expression of FADS2 in bladder cancer tissues (Figure 1D).

### 3.2 FADS2 Can Serve as a Prognostic Indicator in Bladder Cancer

**FIGURE 2:**
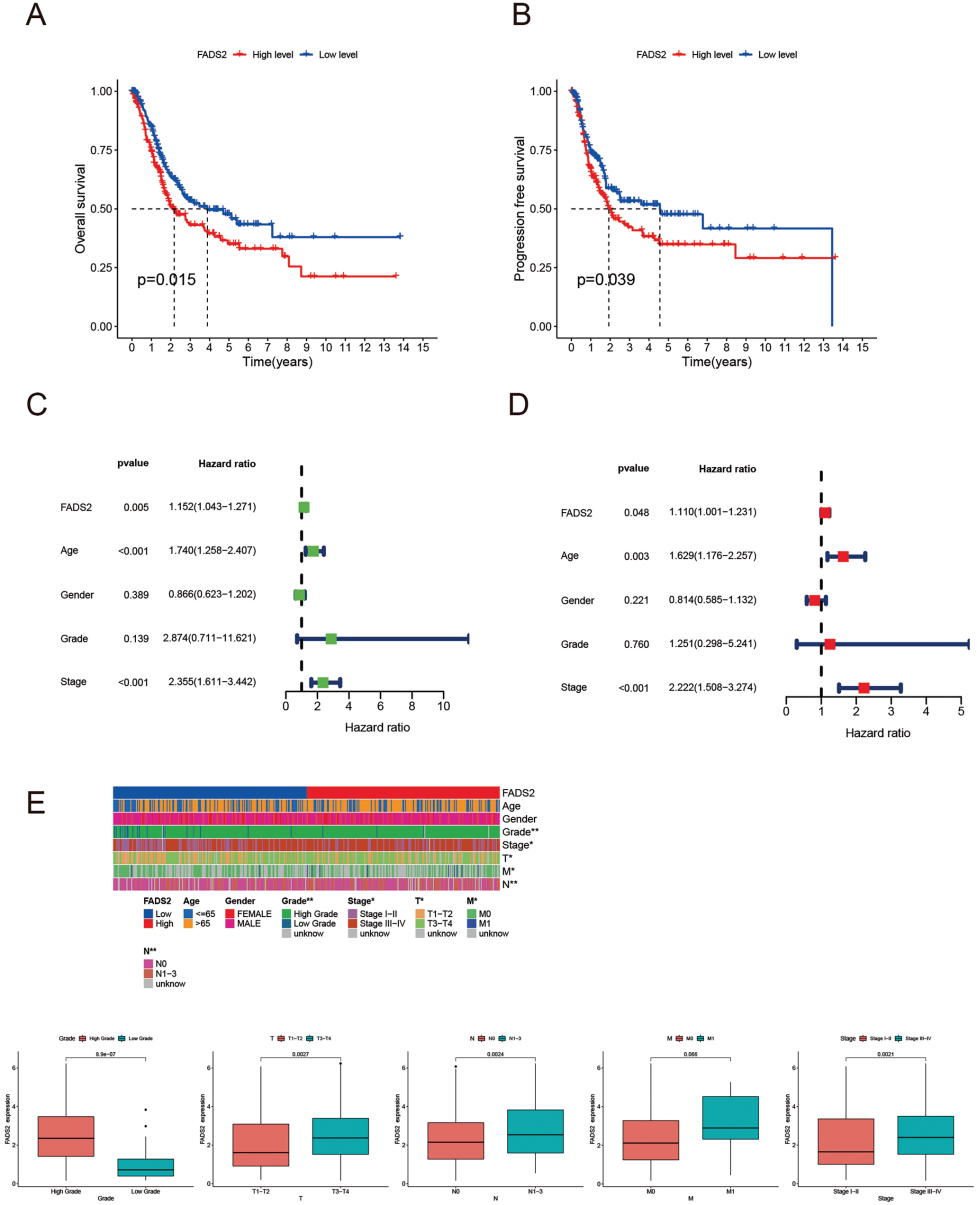
FADS2 serves as a prognostic indicator for bladder cancer. (A) Kaplan–Meier survival analysis of FADS2 in bladder cancer with respect to overall survival. (B) Kaplan–Meier survival analysis of FADS2 in bladder cancer with respect to progression‐free survival. (C) Univariate Cox regression analysis of FADS2 in bladder cancer. (D) Multivariate Cox regression analysis of FADS2 in bladder cancer. (E) Clinical relevance analysis of FADS2 in bladder cancer.

Building on the observation of FADS2's differential expression, its prognostic relevance in bladder cancer was further evaluated. Kaplan–Meier survival analysis revealed a correlation between elevated FADS2 expression and poorer overall survival and progression‐free survival (Figure 2A,B). Additionally, univariate and multivariate Cox regression analyses demonstrated that FADS2 expression serves as an independent predictor of adverse prognosis in patients with bladder cancer (Figure 2C,D). Elevated FADS2 expression was also associated with higher TNM staging and more advanced clinical grading (Figure 2E). Collectively, these findings support the potential of FADS2 as a valuable prognostic biomarker in bladder cancer.

### 3.3 Knocking Down FADS2 Inhibits the Proliferation and Migration Abilities of Bladder Cancer Cells In Vitro

**FIGURE 3:**
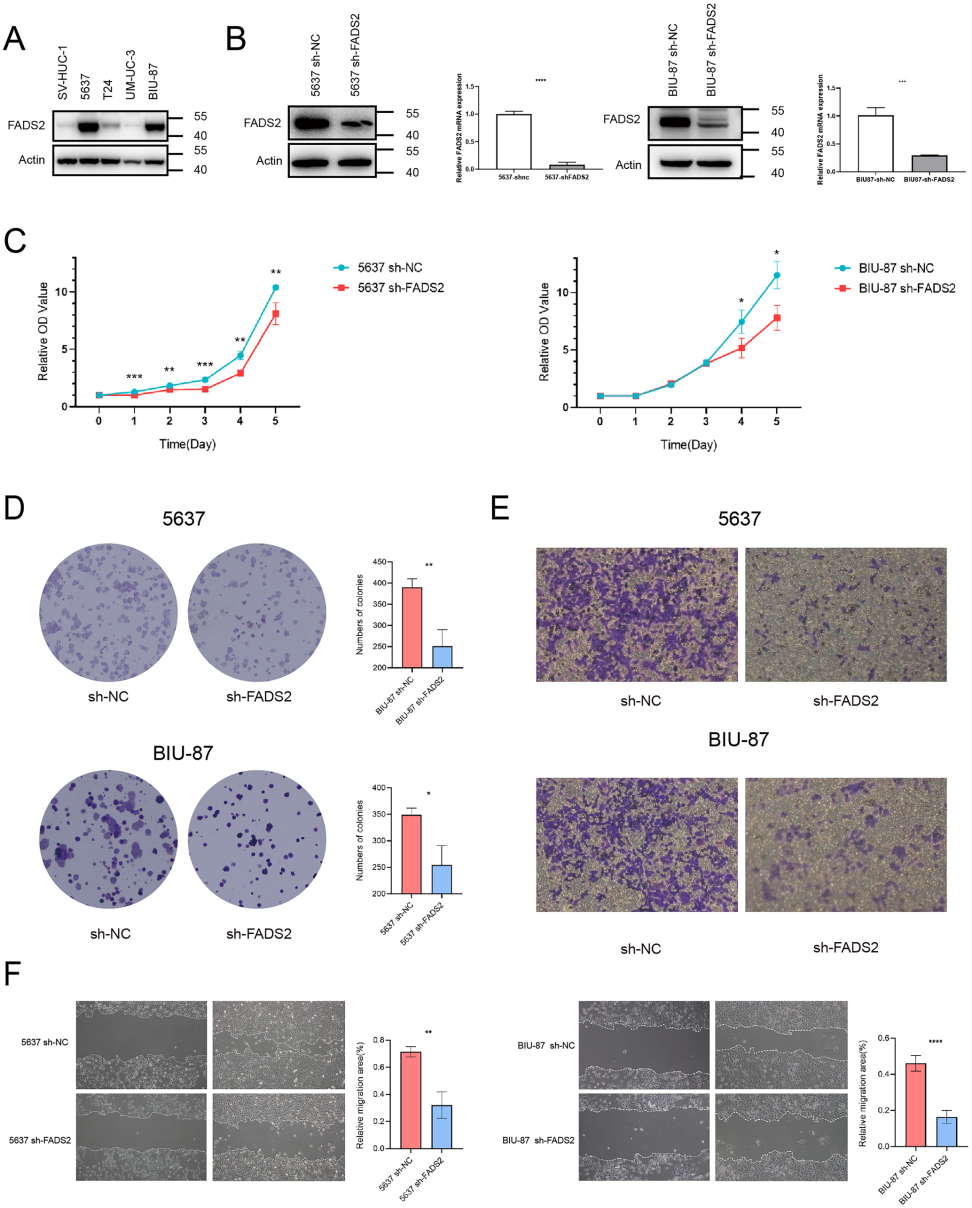
FADS2 influences proliferation and migration abilities of bladder cancer in vitro. (A) Expression of FADS2 in SV‐HUC‐1 and bladder cancer cell lines. (B) FADS2 knockdown in 5637 and BIU‐87 cells. (C) CCK‐8 assay results for 5637 and BIU‐87 cells. (D) Colony formation assay results for 5637 and BIU‐87 cells. (E) Transwell migration assay results for 5637 and BIU‐87 cells. (F) Wound healing assay results for 5637 and BIU‐87 cells. Data are presented as means ± SD (*n* = 3). *p ≤ 0.05; **p ≤ 0.01; ***p ≤ 0.001; ****p ≤ 0.0001.

To investigate the functional role of FADS2 in bladder cancer, a series of in vitro experiments were conducted using FADS2 knockdown cells. Initial analysis revealed low FADS2 expression in SV‐HUC‐1, a normal human ureteral epithelial cell line, and high expression in bladder cancer cell lines, particularly 5637 and BIU‐87 (Figure 31,A,A,S). Consequently, FADS2 knockdown plasmids were transfected into 5637 and BIU‐87 cells to establish bladder cancer cells with reduced FADS2 expression, and the transfection efficiency was confirmed (Figure 3B, S1B). To assess the impact of FADS2 on cell proliferation, CCK‐8 assays were performed, demonstrating a significant decrease in proliferation in the FADS2 knockdown group compared to the sh‐NC group (Figure 3C). Colony formation assays further showed that the FADS2 knockdown group formed fewer and smaller colonies (Figure 3D), collectively indicating that decreased FADS2 expression suppresses bladder cancer cell proliferation. Additionally, to evaluate the effect of FADS2 on cell migration, transwell migration and wound healing assays were conducted. Both assays revealed that FADS2 knockdown in 5637 and BIU‐87 cells significantly reduced their migratory capacity compared to control groups (Figure 3E,F). These results suggest that FADS2 plays a pivotal role in promoting bladder cancer cell proliferation and migration.

### 3.4 Knocking Down FADS2 Can Induce Ferroptosis in Bladder Cancer In Vitro

**FIGURE 4:**
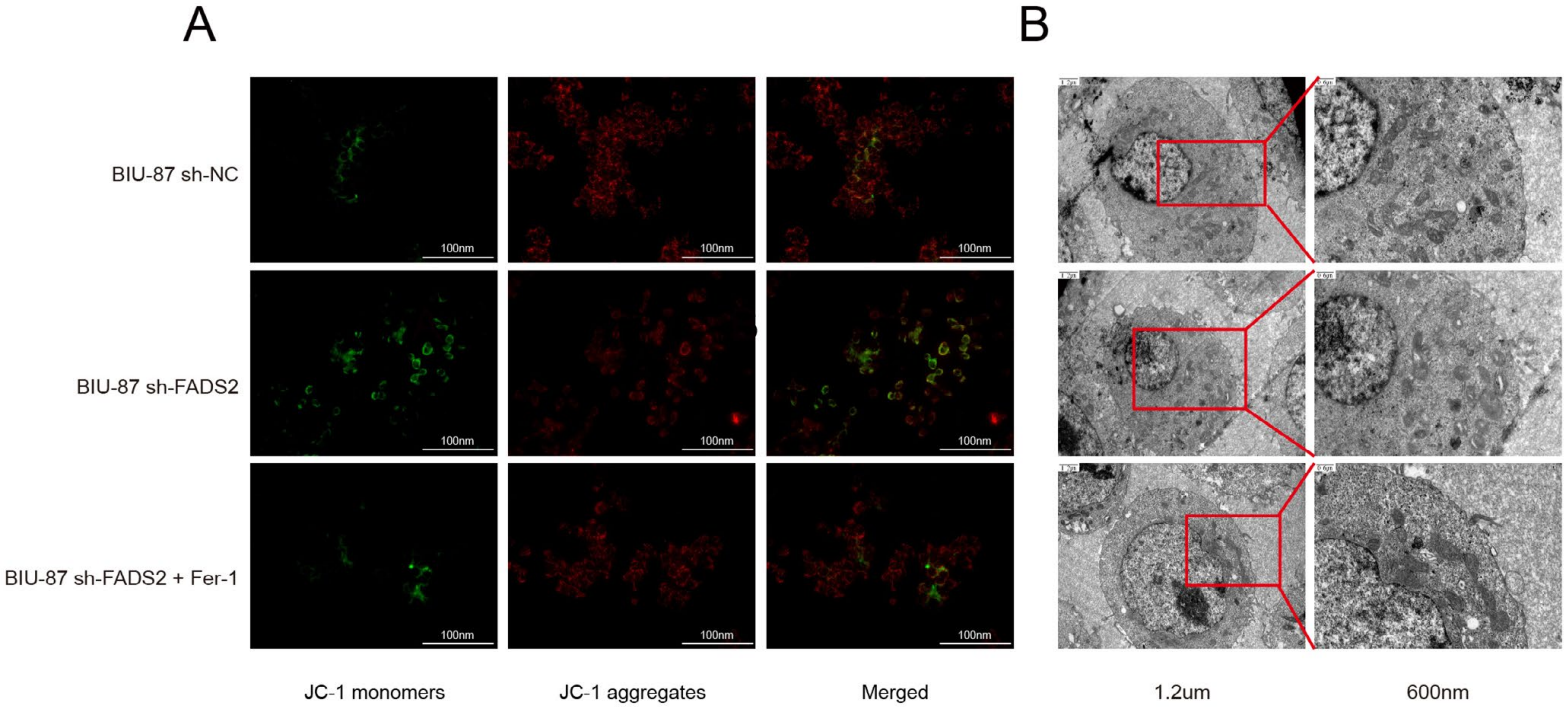
Mitochondrial membrane potential and morphology in FADS2 knockdown cells. (A) Mitochondrial membrane potential staining in vitro. FADS2 knockdown decreases mitochondrial membrane potential, which is reversed by ferrostatin‐1 treatment. (B) Transmission electron microscopy of intracellular mitochondria. FADS2 knockdown causes rupture of the mitochondrial outer membrane and loss of mitochondrial cristae, which is reversed by ferrostatin‐1.

In previous studies, FADS2 was identified as a gene associated with ferroptosis in bladder cancer [27, 28]. Ferroptosis is a distinct form of programmed cell death, characterised by specific subcellular morphological alterations in mitochondria. Mitochondrial membrane potential staining and transmission electron microscopy revealed that FADS2 knockdown in BIU‐87 cells led to a decrease in mitochondrial membrane potential (Figures 4A and S2), rupture of the mitochondrial outer membrane, and a reduction in mitochondrial cristae (Figure 4B) compared to the control group. However, when 2 μM of the ferroptosis inhibitor ferrostatin‐1 was added to FADS2‐knockdown BIU‐87 cells for 24 h, no significant changes in mitochondrial membrane potential or morphology were observed compared to the control group. These results suggest that the ferroptosis inhibitor ferrostatin‐1 can reverse mitochondrial damage induced by reduced FADS2 expression.

**FIGURE 5:**
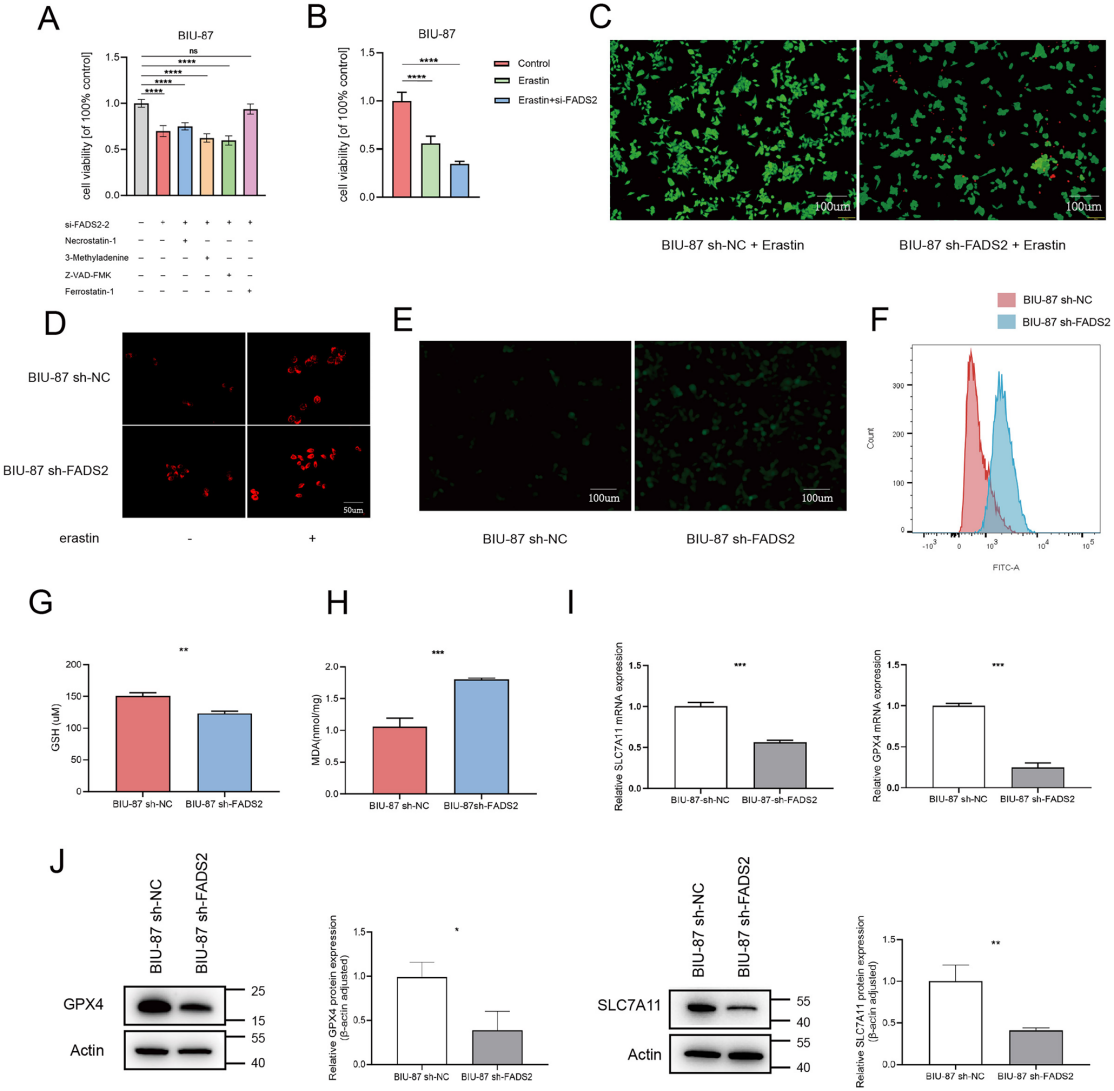
FADS2 knockdown induces ferroptosis in bladder cancer in vitro. (A) Relative cell viability of BIU‐87 cells measured by CCK‐8 assay after incubation with different inhibitors for 24 h. (B) FADS2 knockdown enhances cell viability inhibition upon erastin treatment. (C) Fluorescence images of live/dead staining in BIU‐87 cells after 24‐h treatment with erastin. Scale bars, 100 μm. (D) Iron accumulation detected using the FerroOrange probe. (E) Intracellular total ROS levels measured using the DCFH‐DA probe in BIU‐87 cells. Scale bars, 100 μm. (F) Lipid ROS levels assessed using the C11‐BODIPY probe. (G‐H) GSH depletion and MDA production measured in BIU‐87 cells. (I‐J) Expression of SLC7A11 and GPX4 in BIU‐87 cells. Data are presented as means ± SD (*n* = 3). ***p ≤ 0.001.

To investigate whether FADS2 expression influences ferroptosis, si‐RNA was synthesised to transiently knock down FADS2 expression in BIU‐87 cells (Figure S3), and the effects of various inhibitors on cell viability were assessed (Figure 5A). The results showed that FADS2 knockdown reduced cell viability, an effect that was reversed by a ferroptosis inhibitor, while inhibitors of necrosis, apoptosis and autophagy had no impact. Additionally, cell viability in BIU‐87 cells decreased significantly after erastin treatment and was further reduced following FADS2 knockdown (Figure 5B). A live/dead viability/cytotoxicity assay confirmed that FADS2 knockdown enhanced the growth‐inhibitory effect of erastin on BIU‐87 cells (Figure 5C). Ferroptosis is characterised by elevated lipid peroxidation and iron accumulation [30]. To examine these aspects, the FerroOrange probe was used to detect iron accumulation, and the DCFH‐DA and BODIPY‐C11 probes were utilised to measure total ROS and lipid ROS levels. Increased iron accumulation (Figures 5D and S4), total ROS (Figure 5E) and lipid ROS (Figures 5F and S5) were observed in FADS2‐knockdown BIU‐87 cells. Furthermore, GSH depletion and MDA accumulation, key indicators of lipid peroxidation, were measured. FADS2 knockdown led to a significant increase in both GSH depletion and MDA accumulation compared to the control group (Figure 5G,H), confirming a marked increase in lipid ROS levels following FADS2 inhibition in bladder cancer cells. Additionally, the expression of ferroptosis‐related genes was assessed at both the mRNA and protein levels. Silencing FADS2 resulted in a reduction of SLC7A11 and GPX4 expression (Figure 5I,J). Collectively, these findings suggest that FADS2 plays a role in ferroptosis resistance in bladder cancer and that FADS2 knockdown enhances the sensitivity of bladder cancer cells to ferroptosis.

### 3.5 Inhibition of the mTOR Pathway and SREBP Activity Can Lead to Downregulation of FADS2 Expression and Induce Ferroptosis in Bladder Cancer Cells

**FIGURE 6:**
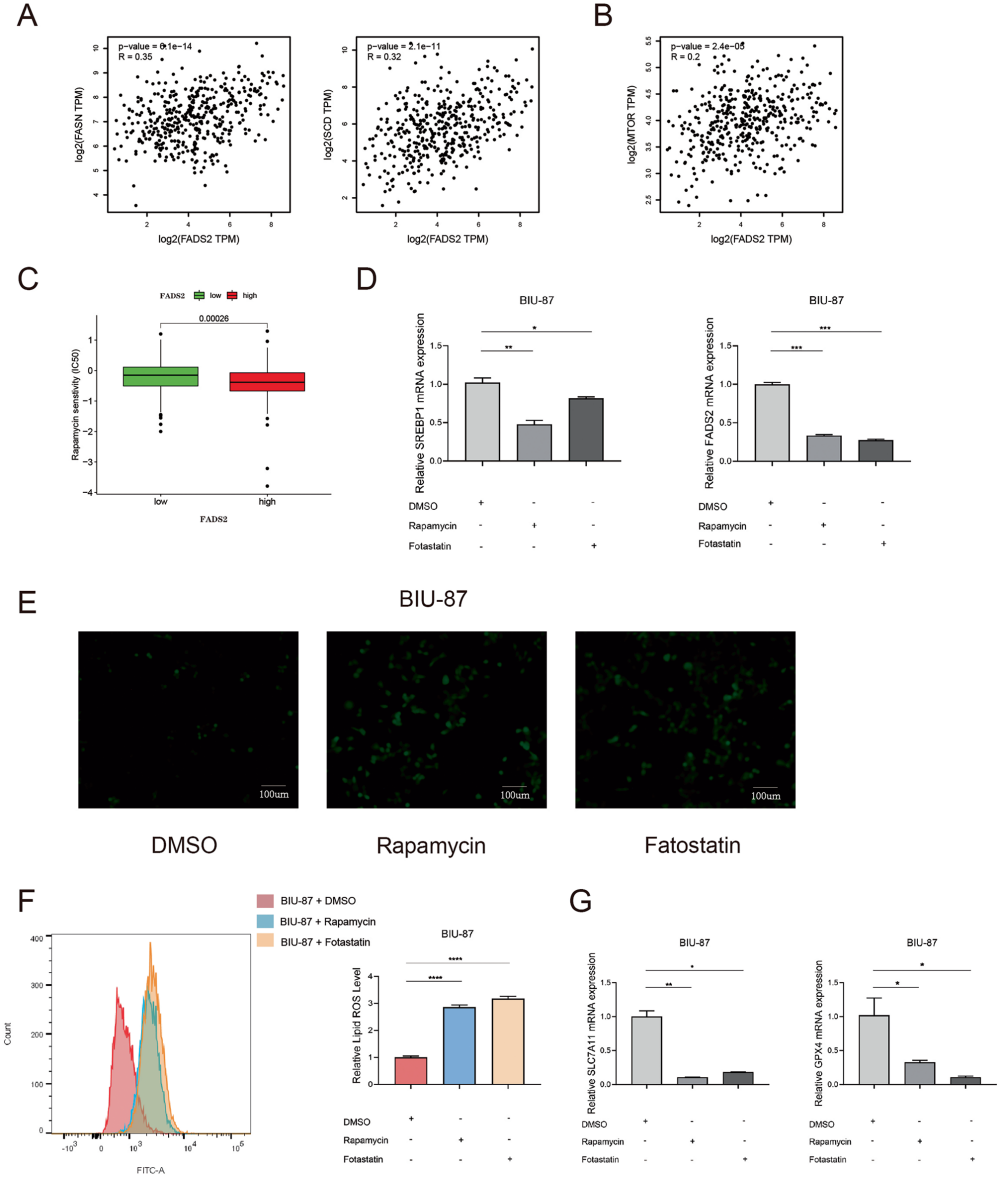
Inhibition of the mTOR pathway and SREBP activity leads to downregulation of FADS2 expression and induces ferroptosis in bladder cancer cells. (A) Correlation between FADS2 mRNA expression and SREBP‐1 targets (SCD1 and FASN) in bladder cancer. (B) Correlation between FADS2 mRNA expression and mTOR in bladder cancer. (C) Sensitivity of patients with bladder cancer to rapamycin based on FADS2 expression. (D) Expression of FADS2 and SREBP1 in BIU‐87 cells treated with DMSO, rapamycin and fatostatin. (E) Intracellular ROS levels measured using the DCFH‐DA probe in BIU‐87 cells treated with DMSO, rapamycin and fatostatin. Scale bars, 100 μm. (F) Lipid ROS levels measured using the C11‐BODIPY 581/591 probe in BIU‐87 cells treated with DMSO, rapamycin and fatostatin. (G) Expression of SLC7A11 and GPX4 in BIU‐87 cells treated with DMSO, rapamycin and fatostatin. Data are presented as means ± SD (*n* = 3). *p ≤ 0.05; **p ≤ 0.01; ***p ≤ 0.001.

Analysis of the TCGA database revealed a significant positive correlation between FADS2 expression in bladder cancer and the activity markers SCD and FASN, which reflect SREBP activity [31, 32] (Figure 6A). These findings suggest that FADS2 expression is regulated by SREBP, a conclusion supported by previous studies [33]. Furthermore, a significant positive correlation was observed between mTOR and FADS2 expression (Figure 6B). Notably, patients with high FADS2 expression showed increased sensitivity to the mTOR inhibitor rapamycin (Figure 6C), implying that FADS2 is regulated by the mTOR signalling pathway. This led to the hypothesis that activation of the mTOR pathway promotes FADS2 overexpression by enhancing SREBP activity, thereby contributing to the resistance of bladder cancer cells to ferroptosis. To test this hypothesis, BIU‐87 cells were treated with the mTOR inhibitor rapamycin and the SREBP inhibitor fatostatin. qRT‐PCR results revealed that mRNA levels of FADS2 and SREBP1 were significantly reduced compared to the DMSO control group (Figure 6D), indicating that inhibition of the mTOR pathway diminishes SREBP1 activity and reduces FADS2 expression. Further analysis of total intracellular ROS (Figure 6E), lipid ROS (Figure 6F) and the expression of ferroptosis‐related genes SLC7A11 and GPX4 in BIU‐87 cells treated with mTOR and SREBP inhibitors (Figure 6G) showed results consistent with those observed in FADS2‐knockdown BIU‐87 cells. These findings suggest that inhibiting the mTOR pathway and SREBP activity can induce ferroptosis in bladder cancer cells. Together, the results indicate that the mTOR–SREBP–DS2 axis plays a pivotal role in ferroptosis resistance in bladder cancer.

### 3.6 Knocking Down FADS2 Inhibits Bladder Cancer Growth In Vivo by Inducing Ferroptosis

**FIGURE 7:**
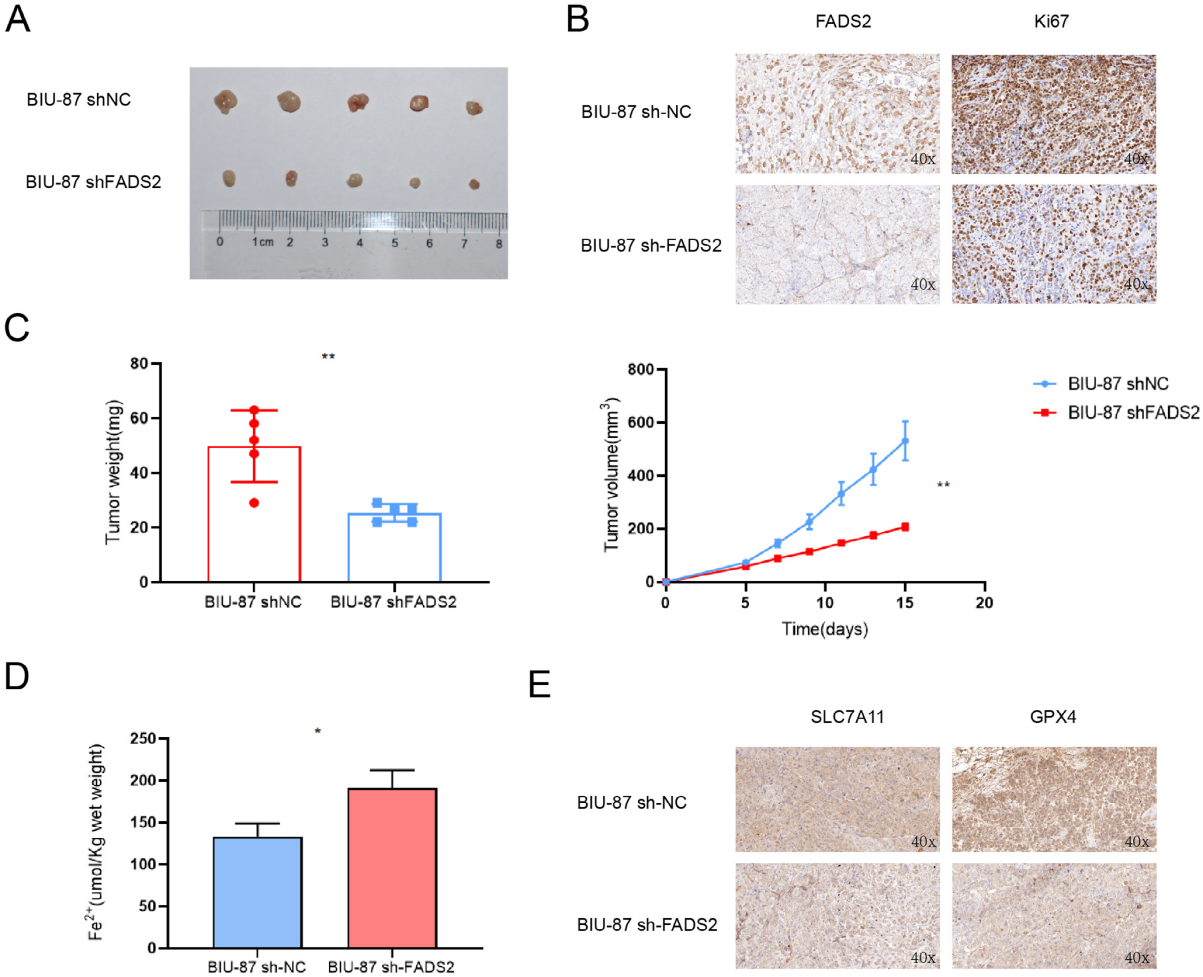
Knocking down FADS2 inhibits bladder cancer growth in vivo by inducing ferroptosis. (A) Tumour tissues from xenograft models established by subcutaneously transplanting BIU‐87 cells into the axilla of mice. (B) Immunohistochemical staining of FADS2 and Ki67 in tumour tissues. (C) Tumour weight (mg) and volume (mm^3^). (D) Measurement of Fe^2+^ production in tumour tissues. (E) Immunohistochemical staining of SLC7A11 and GPX4 in tumour tissues. Data are presented as means ± SD (*n* = 3). *p ≤ 0.05; **p ≤ 0.01.

A xenograft model was established by subcutaneously transplanting BIU‐87 bladder tumour cells to validate the in vitro findings. The results revealed that FADS2 knockdown significantly reduced both the weight and volume of the tumours compared to the control group (Figure 7A,C). Ki67 immunohistochemical staining further confirmed that FADS2 knockdown inhibited bladder cancer growth in vivo (Figure 7B). Given the essential role of iron in ferroptosis induction [5], PUFAs in the cell membrane are prone to peroxidation in the presence of free ferrous ions, leading to the formation of PUFA lipid peroxides. These lipid peroxides accumulate in the cell membrane, causing damage that subsequently triggers ferroptosis. To investigate whether FADS2 knockdown could induce ferroptosis in vivo, ferrous ion content (Figure 7D) and the expression of SLC7A11 and GPX4 were measured by immunohistochemistry (Figure 7E) in mouse tumours. The results showed that reduced FADS2 expression led to the release of ferrous ions in tumour tissues, thereby enhancing the sensitivity of bladder tumours to ferroptosis. Overall, these in vivo findings support the in vitro data, demonstrating that FADS2 knockdown inhibits bladder cancer growth by promoting ferroptosis.

## 4 Discussion

Ferroptosis, a form of programmed cell death, has garnered significant attention in medical biology, particularly in cancer research. Researchers are exploring the potential of inducing ferroptosis as a novel therapeutic approach for cancer treatment, especially for cases resistant to conventional therapies. Ghoochani et al. demonstrated that ferroptosis inducers (FINs) can inhibit the progression of various forms of prostate cancer, including androgen‐sensitive and castration‐resistant prostate cancer. However, during tumour progression, cancer cells develop several mechanisms to resist ferroptosis, such as enhancing antioxidant capacity or inhibiting lipoxygenase activity [34, 35, 36]. Therefore, identifying key molecules involved in ferroptosis and understanding the mechanisms underlying ferroptosis resistance are crucial.

In this study, the expression of FADS2 in tumours was analysed using the TCGA public database. Pan‐cancer analysis revealed that FADS2 was highly expressed in most cancers. Paired differential analysis showed that FADS2 was significantly overexpressed in bladder cancer compared to normal bladder tissue. This finding was further validated using the HPA database and immunohistochemical staining of patient samples from our centre. To assess the prognostic value of FADS2 in bladder cancer, survival analysis indicated that higher FADS2 expression correlates with poorer prognosis. Moreover, Cox regression analysis suggested that FADS2 could serve as an independent prognostic factor for bladder cancer. Clinical correlation analysis revealed that FADS2 expression increased with the advancement of bladder cancer stage, reinforcing its potential as a prognostic marker.

To explore the role of FADS2 in ferroptosis within bladder tumours, FADS2 was knocked down in 5637 and BIU‐87 bladder cancer cells. In vitro, CCK‐8 and colony formation assays showed that silencing FADS2 inhibited bladder tumour growth, while migration and scratch assays demonstrated that FADS2 knockdown reduced the migration capacity of bladder cancer cells. In vivo, a mouse xenograft model confirmed that tumour volume and weight were significantly reduced when FADS2 expression was low. Furthermore, Ki67 immunohistochemical staining revealed a positive correlation between FADS2 expression and tumour malignancy, further supporting the prognostic significance of FADS2 in bladder cancer.

Building on previous findings, FADS2 is implicated in ferroptosis resistance in bladder cancer. This study aimed to further investigate the relationship between FADS2 expression and ferroptosis in bladder tumours. Both in vitro and in vivo, FADS2 knockdown inhibited the proliferation of bladder cancer cells. Upon applying various cell death inhibitors, only the ferroptosis inhibitor reversed the growth inhibition caused by FADS2 knockdown. Additionally, FADS2 knockdown enhanced the sensitivity of bladder cancer cells to FINs, suggesting that elevated FADS2 expression contributes to ferroptosis resistance in bladder cancer. To explore the underlying mechanisms, mitochondrial membrane potential staining and transmission electron microscopy were employed to examine cellular mitochondria. After FADS2 knockdown, mitochondrial changes characteristic of ferroptosis were observed, which could be reversed by the ferroptosis inhibitor Ferrostatin‐1. Moreover, free ferrous ions play a pivotal role in the peroxidation of PUFAs in the cell membrane, leading to membrane damage and ferroptosis induction. Thus, the cellular ferrous ion content is critical during ferroptosis. In the xenograft model, measurements of ferrous ion levels in tumour tissues revealed a significant increase following FADS2 knockdown, further supporting the hypothesis that reduced FADS2 expression promotes ferroptosis. Lipid peroxidation, a hallmark of ferroptosis, was also assessed by measuring GSH, MDA and lipid ROS levels in cells. The increased total ROS observed after FADS2 knockdown was primarily attributed to lipid peroxidation. Collectively, these results demonstrate that FADS2 knockdown enhances oxidative stress in bladder tumours, promoting ferroptosis. In conclusion, these findings underscore the critical role of FADS2 in ferroptosis resistance and suggest that its inhibition could increase the sensitivity of bladder cancer cells to ferroptosis.

FADS2 regulates ferroptosis in bladder cancer by influencing the expression of GPX4 and SLC7A11, but the upstream regulators of FADS2 remain to be explored. Bioinformatics analysis of bladder cancer transcriptome data from the TCGA database revealed a significant positive correlation between FADS2 expression and SCD and FASN, molecules that reflect SREBP activity [32]. Additionally, FADS2 is regulated by SREBP in various tumours. The mTOR signalling pathway, known to regulate SREBP, has been extensively validated. Notably, our analysis showed that patients with bladder cancer exhibiting high FADS2 expression were more sensitive to the mTOR inhibitor rapamycin. Thus, it is hypothesised that mTOR activation upregulates SREBP activity, which in turn promotes FADS2 overexpression, contributing to ferroptosis resistance in bladder cancer cells and facilitating cancer progression. To test this hypothesis, BIU‐87 cells were treated with mTOR and SREBP inhibitors. qRT‐PCR results confirmed that FADS2 is downstream of both mTOR and SREBP. Similar to FADS2 silencing, inhibiting mTOR or SREBP also promoted ferroptosis.

Currently, drug treatments for bladder cancer predominantly involve chemotherapy and immunotherapy. Traditional therapies like cisplatin and anti‐PD‐L1 antibodies also induce ferroptosis [37, 38], but ferroptosis escape may contribute to treatment failure. Our future research will focus on whether inhibiting FADS2 expression can enhance the efficacy of chemotherapy and immunotherapy in bladder cancer.

In conclusion, this study highlights FADS2 as a key player in ferroptosis escape in bladder cancer and unveils a novel mechanism underlying this resistance. These findings offer new insights for therapeutic targeting and provide potential prognostic biomarkers for bladder cancer treatment.

## Author Contributions

**Peixin Li:** conceptualization (equal), resources (lead), writing – original draft (lead). **Shengwen Yao:** methodology (equal), writing – review and editing (equal). **Wenqiang Qi:** software (lead). **Hanwen Liu:** methodology (equal). **Xiaoyi Zhang:** formal analysis (lead). **Bin Zhou:** investigation (lead). **Shijie Zhang:** validation (equal). **Yaozhong Zhang:** validation (equal). **Hao Liang:** investigation (equal). **Huangwei Huang:** data curation (lead). **Yihao Zhao:** visualization (equal). **Benkang Shi:** funding acquisition (lead). **Jun Chen:** project administration (equal), supervision (lead). **Jingchao Liu:** conceptualization (lead).

## Ethics Statement

The collection of patient tissue specimens and the animal study protocol were approved by the Ethics Committee of Qilu Hospital of Shandong University (approval number: KYLL‐2024ZM‐464, dated 29 February 2024). All clinical samples used in this study were obtained with the informed consent of the patients.

## Consent

Written informed consent for publication was obtained from all participants.

## Conflicts of Interest

The authors declare no conflicts of interest.

## Supporting information


**Figure S1A.** FADS2 protein expression in SV‐HUC‐1 and bladder cancer cell lines. Figure S1B: FADS2 knockdown in 5637 and BIU‐87 cells. Data are presented as means ± SD (*n* = 3). *p ≤ 0.05; **p ≤ 0.01; ***p ≤ 0.001.

**Figure S2.** Average fluorescence intensity ratio (Red/Green) in BIU‐87. Data are presented as means ± SD (*n* = 3). ***p ≤ 0.001.

**Figure S3.** The knockdown efficiency of si‐FADS2. Data are presented as means ± SD (*n* = 3). **p ≤ 0.01; ***p ≤ 0.001.

**Figure S4.** Relative FerroOrange fluorescence intensity in BIU‐87. Data are presented as means ± SD (*n* = 3). ***p ≤ 0.001.

**Figure S5.** Relative C11‐BODIPY fluorescence intensity in BIU‐87. Data are presented as means ± SD (*n* = 3). ***p ≤ 0.001.

## Data Availability

The data that support the findings of this study are available from the corresponding author upon reasonable request.
