# Supplementary figures and images for: Identification of FADS2 as a Contributor of Ferroptosis Escape in Bladder Cancer
Source: J Cell Mol Med. 2025 Jul 19;29(14):e70710. doi: 10.1111/jcmm.70710 (PMC12274957; doi:10.1111/jcmm.70710)

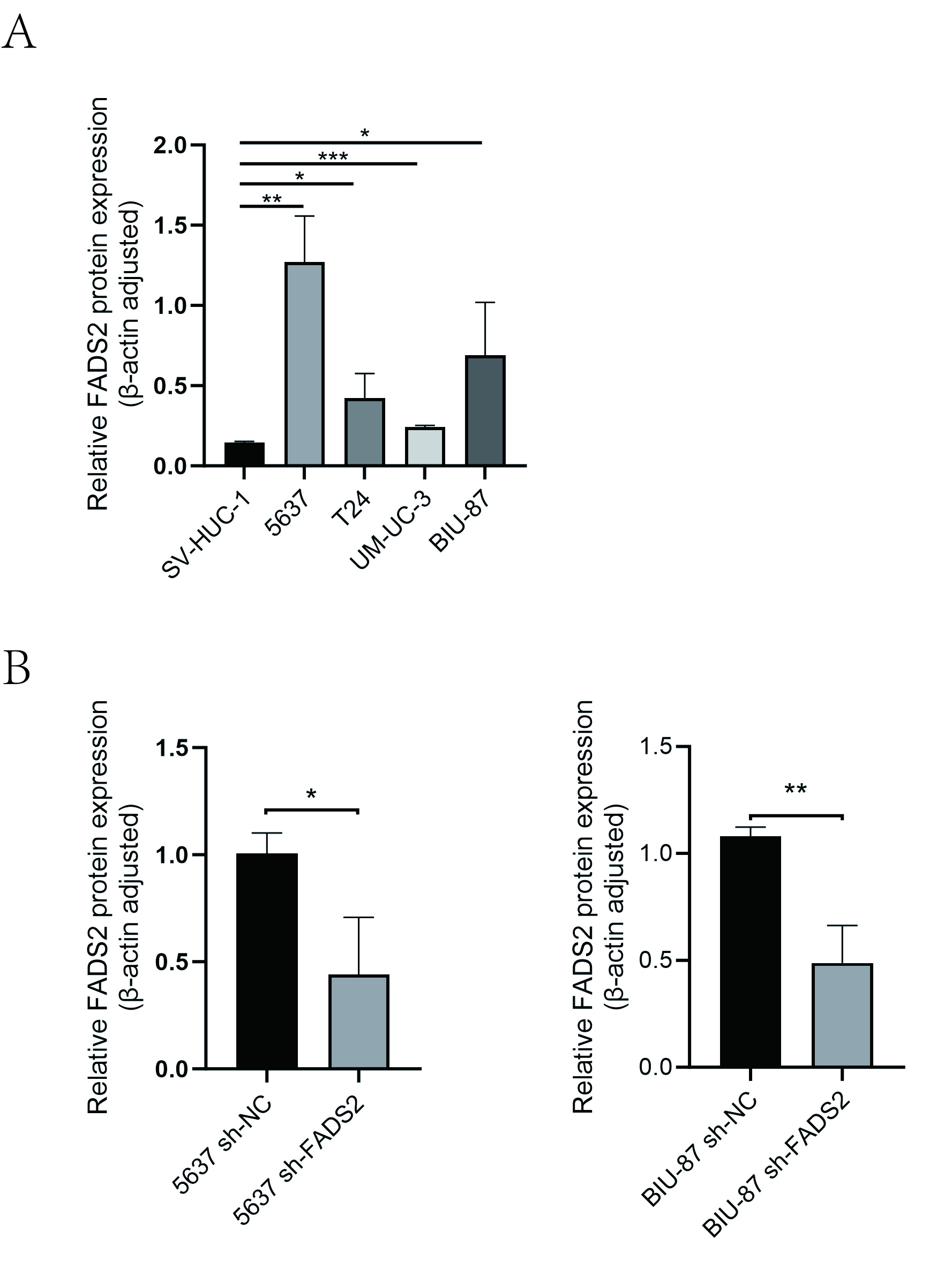

Supplement: Supplementary file 1 — Figure S1A. FADS2 protein expression in SV‐HUC‐1 and bladder cancer cell lines. Figure S1B: FADS2 knockdown in 5637 and BIU‐87 cells. Data are presented as means ± SD (n = 3). *p ≤ 0.05; **p ≤ 0.01; ***p ≤ 0.001. [file JCMM-29-e70710-s004.jpg]

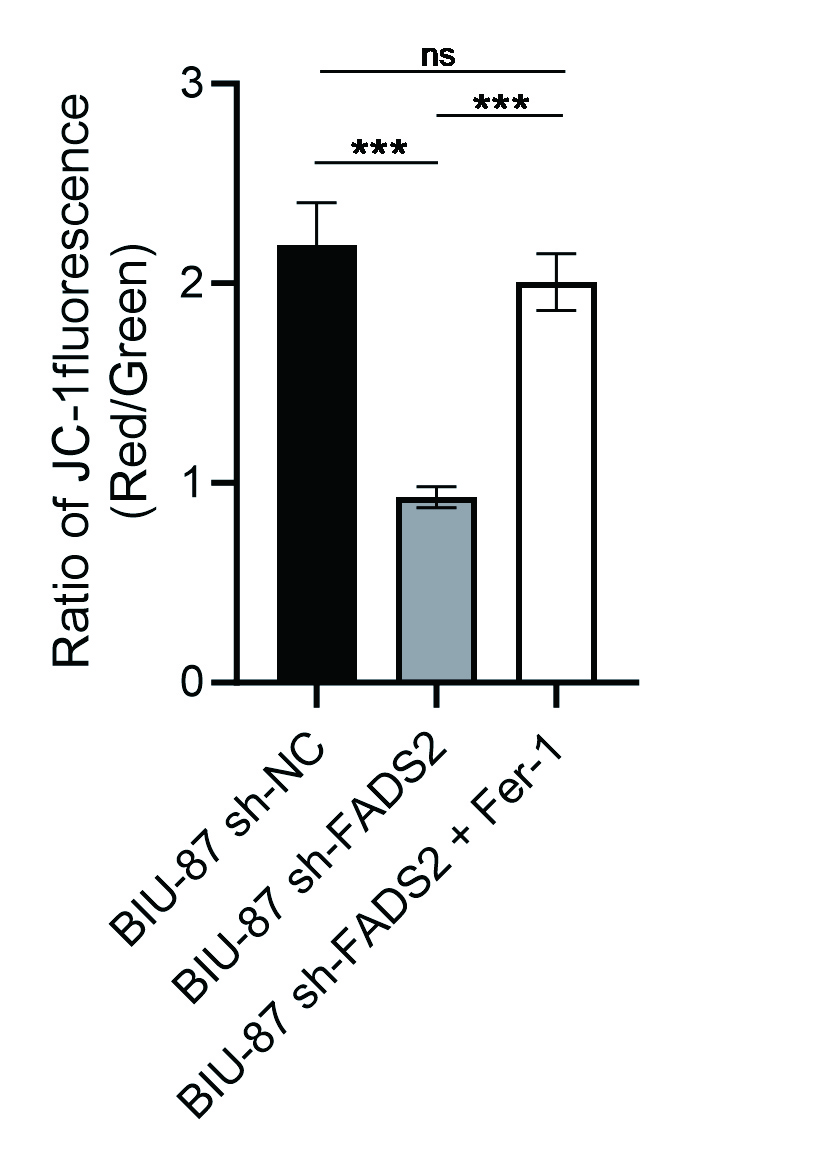

Supplement: Supplementary file 2 — Figure S2. Average fluorescence intensity ratio (Red/Green) in BIU‐87. Data are presented as means ± SD (n = 3). ***p ≤ 0.001. [file JCMM-29-e70710-s002.jpg]

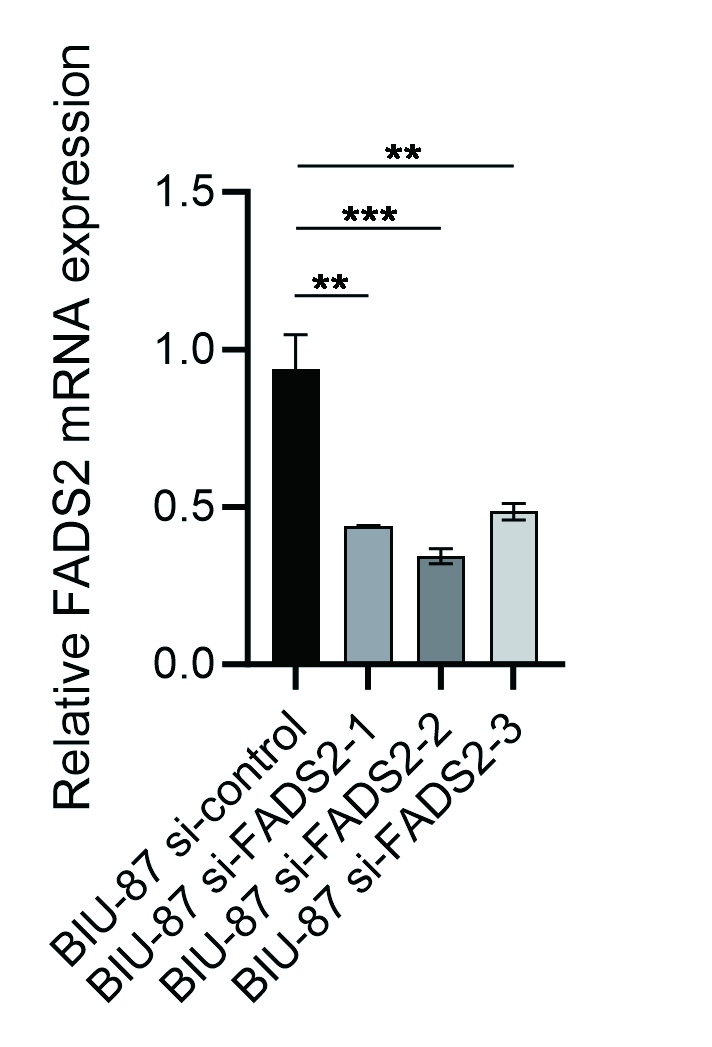

Supplement: Supplementary file 3 — Figure S3. The knockdown efficiency of si‐FADS2. Data are presented as means ± SD (n = 3). **p ≤ 0.01; ***p ≤ 0.001. [file JCMM-29-e70710-s001.jpg]

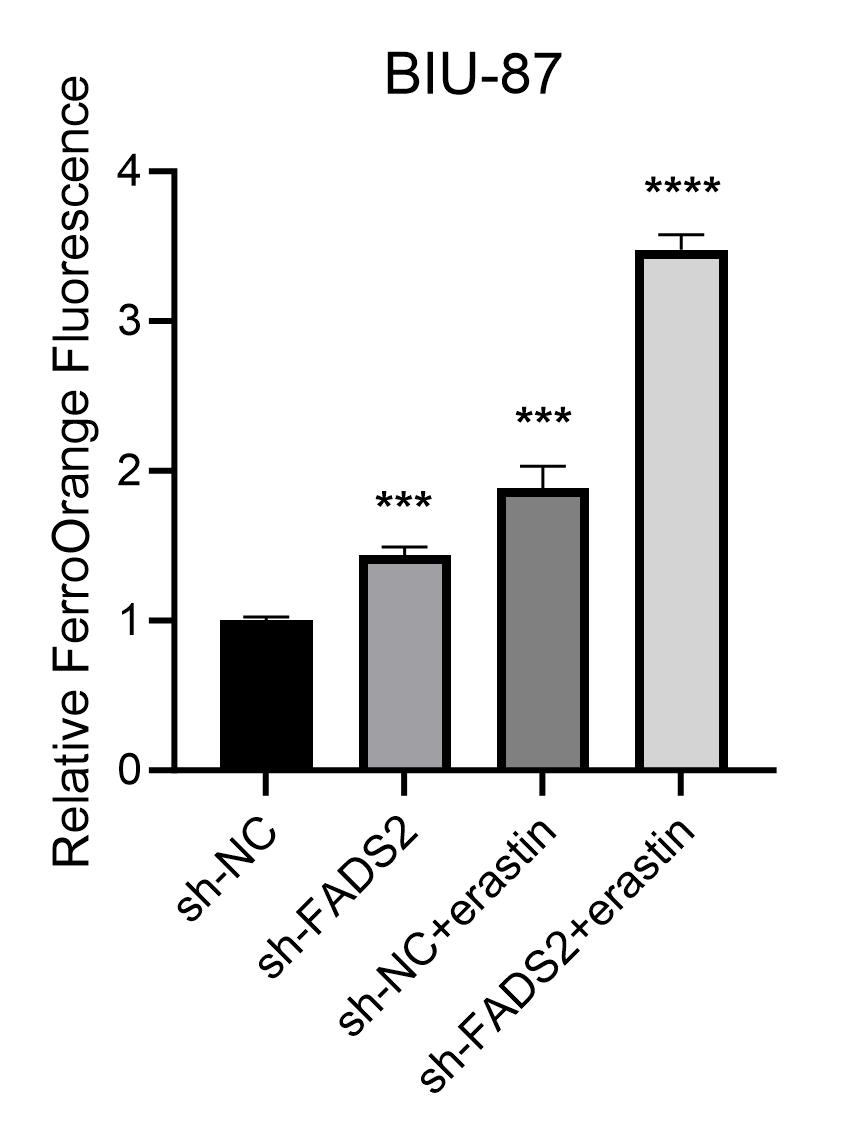

Supplement: Supplementary file 4 — Figure S4. Relative FerroOrange fluorescence intensity in BIU‐87. Data are presented as means ± SD (n = 3). ***p ≤ 0.001. [file JCMM-29-e70710-s005.jpg]

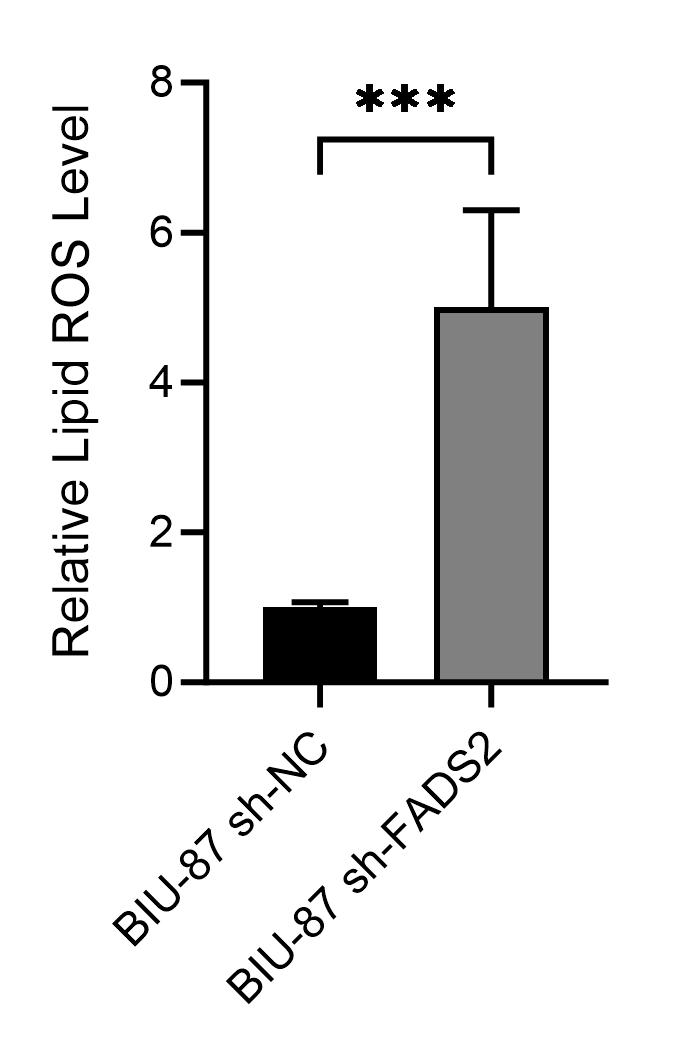

Supplement: Supplementary file 5 — Figure S5. Relative C11‐BODIPY fluorescence intensity in BIU‐87. Data are presented as means ± SD (n = 3). ***p ≤ 0.001. [file JCMM-29-e70710-s003.jpg]
